# Investigating the impact of preslaughter handling intensity on goats: a study on behavior, physiology, blood enzymes, and hormonal responses

**DOI:** 10.3389/fvets.2024.1381806

**Published:** 2024-05-02

**Authors:** Abdullah N. Al-Owaimer, Gamaleldin M. Suliman, Mohsen M. Alobre, Ayman A. Swelum, Mohammed A. Al-Badwi, Hani Ba-Awadh, Awis Qurni Sazili, Pavan Kumar, Ubedullah Kaka

**Affiliations:** ^1^Department of Animal Production, College of Food and Agriculture Sciences, King Saud University, Riyadh, Saudi Arabia; ^2^Halal Products Research Institute, Putra Infoport, Universiti Putra Malaysia, Serdang, Selangor, Malaysia; ^3^Institute of Tropical Agriculture and Food Security, Universiti Putra Malaysia, Serdang, Selangor, Malaysia; ^4^Department of Companion Animal Medicine and Surgery, Faculty of Veterinary Medicine, Universiti Putra Malaysia, Serdang, Selangor, Malaysia

**Keywords:** preslaughter handling, Ardi goat, animal welfare, stress, catecholamines

## Abstract

**Introduction:**

The present study evaluated the effect of preslaughter stress intensities on the behavioral, physiological, blood biochemicals, and hormonal responses in goats.

**Methods:**

Twenty-seven intact male goats (Ardi breed, 10 months of age, 27 kg liveweight) were divided into three treatment groups *viz*., the control (C) group, ear pulling (EP) group, and leg pulling (LP) group. Various behavioral, physiological, blood biochemical and hormonal responses were recorded before and after handling.

**Results and Discussion:**

The EP and LP goats had a higher frequency and intensity of vocalization as compared to control goats. The preslaughter handling stress intensities had a significant effect on the before and after handling values of heart rate, respiration rate, rectal temperature, and ear temperature. Further, among groups, the glucose value increased significantly upon preslaughter handling as compared to the baseline value. The LP goats had significantly higher after-handling value for lactate dehydrogenase (LDH) as compared to the before-handling value. The catecholamines (adrenaline and noradrenaline) and β-endorphin concentrations increased significantly upon preslaughter handling. The higher physiological, behavioral, blood biochemical, and hormonal response indicated higher preslaughter stress in EP and LP goats. Both levels of intensity revealed unfavorable responses in goats that may adversely affect animal welfare and meat quality. Thus, to ensure minimal adverse effects on behavior, physiology, blood enzymes, and hormonal responses, it is recommended to follow animal welfare principles when implementing preslaughter handling practices.

## 1 Introduction

Goat rearing is very common in developing countries in Asia and Africa for meat, milk, and leather production. Goats can be reared with minimum feed and housing inputs, providing extra income and sustenance to poor people (1). Thus, it has a crucial role in the agrarian economy and could be a sustainable source of animal protein in challenging conditions of drought and scarcity of natural resources (2). The goat population in Saud Arabia was estimated to be 6,779,154 in 2022. Ardi goat is the most popular breed reared in the harsh desert conditions of Saudi Arabia (3). It is the largest goat breed in Saudi Arabia and is reared for meat and milk production. It has excellent tolerance to harsh climatic conditions, thriving well and maintaining production even with limited resources (4).

Ardi goats, like other livestock, are inevitably handled during the slaughtering operations. Various preslaughter handling practices and procedures of animals at the farm, during transport and marketing, and at the slaughterhouses may subject the animals to various stressors. The animal’s response to stress is very complex and multivariant and depends upon interactions of several factors such as type, intensity, and duration of stress, genetic makeup, previous exposure, perceptions of stressors, and intra-animal variations (5, 6). The activation of the sympathoadrenal (SPA) and hypothalamic–pituitary–adrenal (HPA) axes under preslaughter stress releases catecholamines (noradrenaline/norepinephrine) secretion, which leads to various physiological and behavioral responses to increased energy demand needed for “flight or fight responses” (7, 8).

These stressors can adversely affect the welfare of animals because of the negative emotions they elicit. To respond to the potential threat perceived, the animals may express different behavioral and physiological reactions (9), such as increasing heartbeat, respiration rate, rectal temperature, plasma cortisol, and catecholamine concentrations (10, 11). These alterations could be used as indicators of stress in animals (11). Exposure to various stressors leads to poor animal welfare and the animals’ physiological reactions and associated metabolic changes adversely affect meat quality (5, 12, 13).

The issue of animal welfare has taken center stage and affected meat production and marketing, with the potential to impact overall meat demand (14–16). With increasing awareness and education, consumers are now more conscious about the welfare of animals and the treatment given to animals throughout the production chain (17–19). Proper compliance with animal welfare during meat production, particularly handling before slaughter, could improve animal productivity and the welfare of livestock. In addition, proper preslaughtering is crucial for ethical meat production and ensuring the spiritual quality of meat (20, 21).

Several studies evaluated the effect of preslaughter handling stress in animals on animal welfare and meat quality (22–24), but there are still limited studies dealing with the handling of goats during the most crucial time of handling from lairage to slaughter point. Furthermore, studies on the preslaughter handling of Ardi goat are still scarce. Thus, the present study was designed to assess the effect of preslaughter handling stress on behavioral, physiological, blood biochemicals, and hormonal responses in Ardi goats. The outcome of the present study would help convince the people involved in the meat industry to follow proper animal handling principles and provide assurance of animal welfare principles.

## 2 Materials and methods

### 2.1 Ethical approval

The Research Ethics Committee (REC) of King Saud University approved the present study under approval number KSU-SE-22-112.

### 2.2 Animals and experimental design

Twenty-seven (27) intact males of the Ardi goat breed, 10 months of age and weighing approximately 27 kg, were used in the present study. The goats were housed at the Research Station of the Department of Animal Production (24°48′22.1”N 46°31′13.4″ E), College of Food and Agricultural Sciences, King Saud University, Saudi Arabia. The goats had a 14-day adaptation period with proper provision of commercial total mixed ration *ad libitum*. The goats had free access to clean, fresh tap water, and regular veterinary services provided by a trained veterinarian.

Before the start of the experiment, the goats were randomly divided into three groups (*n* = 9), *viz.*, C, EP, and LP. The evaluation parameters were measured for all animals in three groups before-handling (BH) and after-handling (AH). The distance between the research station and the slaughterhouse is approximately 15 km, and it takes the vehicle approximately 25 min to reach its destination. Before commencing the experiment, the goats spent approximately 30 min in the holding area.

Control goats were brought and restrained gently with minimum human touch and by following animal welfare principles. Ear-pulling (EP) goats were mobilized through the slaughterhouse by slaughterhouse personnel pulling their ears from the holding area (lairage) until the point of slaughter. Leg-pulling (LP) goats were mobilized through the slaughterhouse with their hind legs pulled by slaughterhouse personnel from the lairage to the point of slaughter. All goats were brought from the same distance and under the same floor conditions. Various behavioral, physiological, blood biochemical, and hormonal responses were recorded before the start of the experiment at the animal holding area (pre-handling stage) and AH at the slaughter point (AH stage).

### 2.3 Recording of behavioral responses

The behavioral responses of Ardi goats during preslaughter handling were recorded manually through video recording. The behavioral responses during preslaughter handling were recorded by two technical staff members who were experts in goat handling and animal welfare. Vocalization was recorded by measuring the number of bleats and their intensity/pitch. Involuntary urination was recorded by counting the number of times the goat urinated during the experiment.

The behavior of goats for vocalization during handling was classified as no, low, medium, or high vocalization. The total percentages of goats showing particular behavioral responses were recorded. The frequency of vocalization per animal was recorded.

### 2.4 Measurement of physiological responses

The physiological responses of goats in the different groups were measured at the lairage BH and at the slaughter point AH by recording heart rate (by stethoscope), respiration rate/ breathing frequency (by using a stopwatch), rectal (by a digital thermometer), ocular, and coat temperature (by infrared thermometer). During the process of data recording and blood collection, goats were restrained minimally, and heart rate measurement and blood collection were performed by experienced technical staff.

### 2.5 Blood collection

The trained technical staff collected blood from the external jugular vein. Briefly, the animals had an indwelling catheter inserted at least 30 min before sample collection for basal samples. The blood was collected into a chilled lithium-heparin tube. Samples were centrifuged at 4°C immediately following collection (not >20 min after collection), and the resulting plasma was frozen immediately. Blood samples were collected from all animals in all groups two times, *viz.*, BH in lairage and at slaughter point AH. The blood samples were collected by using 21-gauge needles connected to 10 mL clot activator (BD Vacutainer^®^, Plymouth, United Kingdom) ethylenediaminetetraacetic acid (EDTA) tubes. The tubes containing collected blood samples were kept upright in a box containing crushed ice for 1 h, followed by refrigerated centrifugation (Eppendorf Centrifuge 5,810) at 3500 *g* for 15 min at 4°C. The plasma obtained was stored in a deep freezer (Sanyo Electric Co Ltd., United Kingdom) at −80°C until subsequent hormonal analysis.

### 2.6 Blood biochemical analysis

The blood biochemical analysis of plasma samples was performed at the Clinical Pathology laboratory, Veterinary Laboratory Service Unit, Faculty of Veterinary Medicine, Universiti Putra Malaysia. The alanine aminotransferase (ALT), glucose, lactate, creatine kinase (CK), and lactate dehydrogenase (LDH) were assessed using a benchtop chemistry analyzer (Biolis 24i Premium & Biosystems BA400, Tokyo Boeki, Japan).

### 2.7 Hormonal analysis

The blood plasma concentrations of catecholamines (adrenaline and nor-adrenaline) and β-endorphin were determined using the highly sensitive adrenaline (BA E-4100), noradrenaline (BA E-4200), and β-endorphin (QY-E140008) enzyme-linked immunoassay kits (ELISA; QAYEE-BIO-Technology Co. Ltd., Shanghai, China). The hormonal analysis was conducted as per the manufacturer’s instructions.

### 2.8 Statistical analysis

The data were presented as mean along with standard error. A paired *t*-test was used to analyze the difference between BH and AH values for physiological and hormonal responses in the same group. All behavior data using Fisher’s exact test except for the frequency of vocalization were analyzed using a one-way analysis of variance (ANOVA, *n* = 9). Responses between groups and handling intensities were compared with Duncan’s multiple-range test using ANOVA. A level of significance (*p*-value) of less than 0.05 was considered statistically significant.

## 3 Results and discussion

### 3.1 Behavioral responses

**Figure 1:**
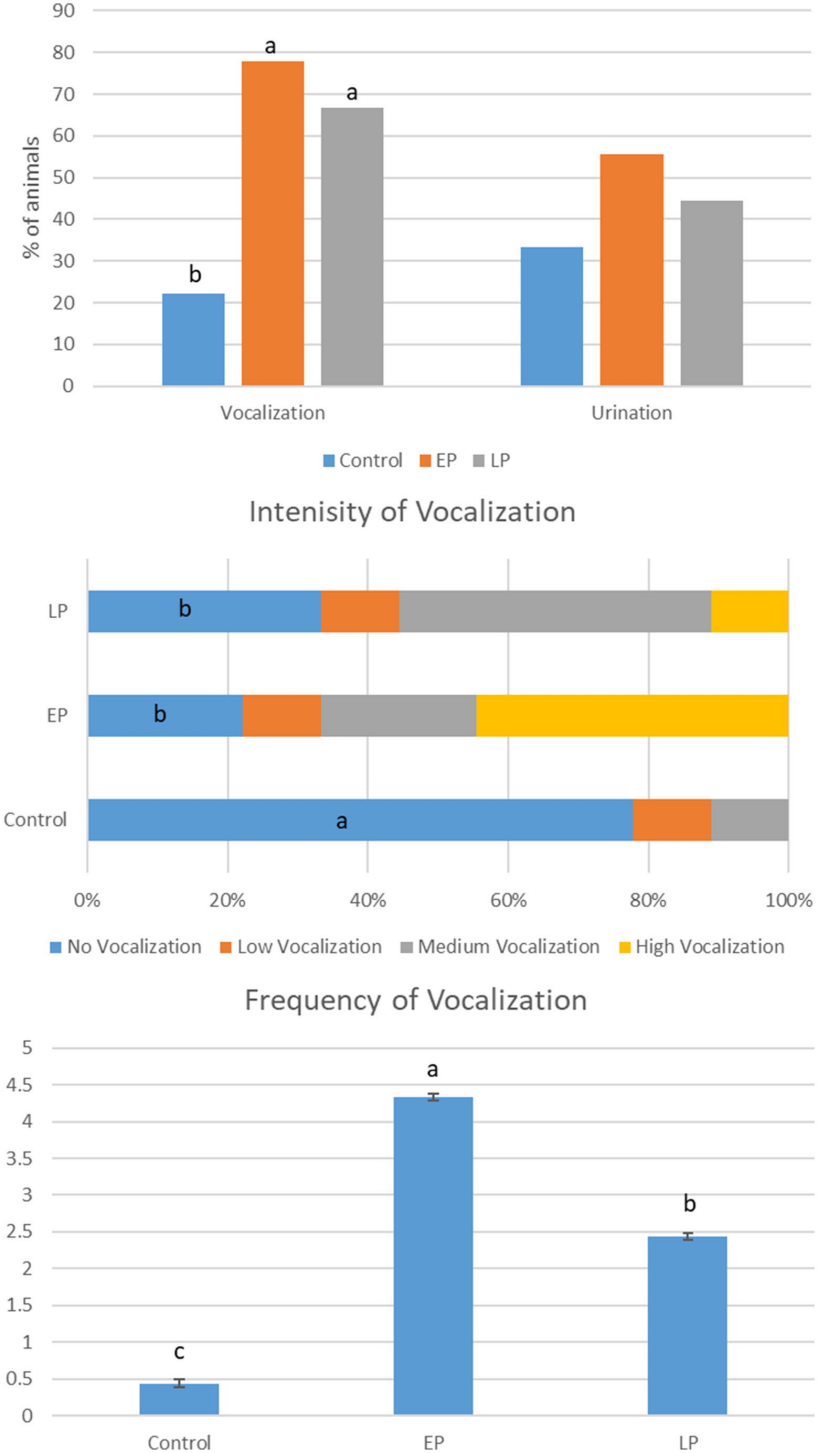
Effects of preslaughter handling intensities on the frequency and scale of vocalization and urination in goats. Values with different small letters, a, b, c differ significantly (*p* < 0.05), Control, goats moved from lairage to slaughter point gently; EP, goats moved from lairage to slaughter point by ear pulling; LP, goats moved from lairage to slaughter point by hindleg pulling, *n* = 9.

Preslaughter handling intensities had a significant effect on the scale of vocalization in Ardi goats (Figure 1). The percentage of goats that vocalized was significantly higher in the EP and LP groups than in the control group. Interestingly, in the present study, a significantly higher frequency of vocalization was recorded for EP goats, followed by LP goats, than the control goats. The control group goats had mild vocalization and a very low level of frequency, with only two out of nine goats recorded to vocalize during the experiment. Involuntary urination was recorded in all three groups of goats without significant differences.

In the present study, vocalization during preslaughter handling in EP and LP goats was observed. This potentially indicates the stress in goats and compromised welfare status. Compared to other farm animals, goats have aggressive behavior, thereby causing significant changes in their behavioral and physiological parameters during handling operations (25). Intense vocalization is correlated with stress or painful conditions in goats and other ruminants (26). The higher intensity of vocalization recorded in EP goats could be due to the stretching of soft and sensitive tissue of the external ear during EP in these goats. Similarly, a higher degree of vocalization in cattle and pig slaughterhouses was linked with poor handling practices, improper restraints, and stunning (27–30). Furthermore, a higher frequency of involuntary urination was reported in stressful conditions due to stretching of the bladder under the activation of the peripheral and central nervous system and the release of pro-micturition molecules (31).

### 3.2 Physiological response

The preslaughter stress intensities resulted in increased physiological responses in goats. The preslaughter handling stress intensities had a significant effect on the before and AH values of heart rate, respiration rate, rectal temperature, and ear temperature (Table 1). Within the group, BH and AH heart rate values were noted to be significantly (*p* = 0.016) different in LP goats. The BH and AH heart rate values did not differ significantly, whereas, in the LP groups, the AH heart rate value was significantly (*p* = 0.016) higher than their respective BH values for animals of the same group. The BH and AH values of respiration rate in the LP group differed significantly (*p* = 0.017), and an increase of 108.8% was noticed. For EP goats, a comparatively lower increase of 49.2% in respiration rate was recorded for the AH value as compared to their BH value. The BH and AH values for rectal temperature had significant differences for EP goats (*p* = 0.019) and LP goats (*p* = 0.038), whereas for the control groups, these values were recorded as comparable (*p* = 0.736). The eye temperature and coat temperature values were noted to be comparable with non-significant differences between their respective BH and AH values for all three groups of goats, *viz.*, C, EP, and LP.

**Table 1:** Effects of pre-slaughter handling intensities on heart rate, respiration rate, and body temperature in goats.

Parameter	Sampling	Control	EP	LP	*p*-value
Heart rate (beats/min)	BH	81.75 ± 1.52	81.09 ± 1.59	81.27 ± 2.50	0.969
	AH	83.83 ± 1.57^a^	130.43 ± 5.23^b^	157.09 ± 8.19^c^	<0.001
	*p*-value	0.490	0.375	0.016	
Respiration rate (breaths/min)	BH	58.09 ± 1.39	58.10 ± 3.83	58.90 ± 3.17	0.976
	AH	62.82 ± 4.63^a^	86.71 ± 5.71^a^	123.08 ± 16.28^b^	0.002
	*p*-value	0.133	0.559	0.017	
Rectal temperature (°C)	BH	39.84 ± 0.23	39.89 ± 0.23	39.84 ± 0.23	0.098
	AH	39.87 ± 0.21^a^	40.38 ± 0.16^ab^	40.44 ± 0.15^b^	0.058
	*p*-value	0.736	0.019	0.038	
Eye temperature (°C)	BH	33.47 ± 0.38	33.42 ± 0.45	33.53 ± 0.33	0.980
	AH	32.06 ± 0.28^a^	33.62 ± 0.38^b^	33.81 ± 0.44^b^	0.005
	*p*-value	0.067	0.929	0.654	
Ear temperature (°C)	BH	25.29 ± 0.34	25.34 ± 0.33	25.59 ± 0.42	0.912
	AH	25.52 ± 0.43^a^	28.44 ± 0.60^b^	27.88 ± 0.67^b^	0.001
	*p*-value	0.822	0.036	0.160	
Coat temperature (°C)	BH	26.24 ± 0.36	26.16 ± 0.36	26.14 ± 0.32	0.859
	AH	26.27 ± 0.43^a^	27.74 ± 0.35^b^	27.53 ± 0.31^b^	0.022
	*p*-value	0.240	0.123	0.339	

Values are mean ± standard error, with different superscripts small letters, a, b, c--within a row differ significantly (*p* < 0.05), BH, value before-handling in lairage; AH, value after-handling at slaughter point; Control, goats moved from lairage to slaughter point gently; EP, goats moved from lairage to slaughter point by ear pulling; LP, goats moved from lairage to slaughter point by hindleg pulling; level of significance- 5% (*p* < 0.05), *n* = 9.

The BH groups did not differ significantly in physiological responses. Significant differences were observed in the AH stages. Specifically, eye, ear, and coat temperatures were higher for the EP and LP groups compared to the control group. Rectal temperatures were higher in LP than in C animals, with EP having intermediate values. Respiration was faster in the LP than in the other groups. Finally, the AH heart rate value was significantly faster in LP than in the EP and C animals and significantly faster in EP than in C animals (LP > EP > C, see Table 1). Upon preslaughter handling, the highest heart rate was recorded for LP goats and followed the following order: LP > EP > Control. The respiration rate of LP goats was recorded as significantly higher than that of EP goats after preslaughter handling. The AH rectal temperature was recorded as the highest for the LP goats, which in turn was comparable to that of EP goats. The AH rectal temperature values did not alter significantly for the control and EP goats. Furthermore, AH values for eye temperature and coat temperature of EP and LP goats were recorded as comparable and significantly higher than the control goats.

The increased physiological responses in the EP and LP goats AH could be due to the activation of SPA under stress, consequently releasing adrenaline from preganglionic nerve terminals and noradrenaline from post-ganglionic nerve terminals of the adrenal medulla (32). This causes higher energy production coupled with various physiological responses needed for higher energy demand, such as increased heart rate, body temperature, and blood pressure, to prepare animals to face stressful conditions (7, 33). In cases where the stressor persists, SPA activation is followed by activation of the HPA axis, thereby releasing ACTH by the anterior pituitary, which in turn releases cortisol from the adrenal cortex into the blood circulation (7, 34). These above changes lead to increased catabolic activities, causing an increase in heart rate, respiration rate, and body temperature, as observed in the present study AH. Furthermore, a higher physiological response in the LP goats as compared to EP goats could be due to the higher stress and pain perception by goats during high-intensity handling (by hind legs) as compared to low-intensity handling (by ear).

### 3.3 Blood glucose and lactate concentrations

The BH and AH values of blood glucose and blood lactate concentrations did not differ significantly among all three groups of goats (Table 2). AH blood glucose concentration increased to 77.49% in the EP group and 80.07% in the LP goats, as compared to their respective handling values of glucose. There were significant interactions between groups and handling intensities for AH blood glucose and blood lactate concentrations. The EP and LP blood glucose concentrations were noted to be comparable and significantly (*p* < 0.05) higher than C after preslaughter handling. The AH blood lactate concentration of the control goats did not differ significantly (*p* > 0.05) from that of the EP goats but had significantly (*p* < 0.05) lower values than those of the EP and LP goats.

**Table 2:** Effects of pre-slaughter handling intensities on blood alanine aminotransferase, glucose, lactate, creatine kinase, and lactate dehydrogenase concentrations in goats.

Parameters	Sampling	Control	LI	LP	*p*-value
ALT (U/L)	BH	17.44 ± 0.63	17.22 ± 0.46	17.00 ± 0.71	0.875
	AH	17.56 ± 0.50	17.78 ± 0.80	17.67 ± 0.47	0.976
	*p*-value	0.223	0.466	0.831	
Glucose (mMol/L)	BH	3.31 ± 0.20	3.51 ± 0.17	3.31 ± 0.13	0.636
	AH	4.61 ± 0.22^a^	6.23 ± 0.57^b^	5.98 ± 0.35^b^	0.021
	*p*-value	0.960	0.147	0.621	
Lactate (mMol/L)	BH	3.57 ± 0.19	3.38 ± 0.35	3.53 ± 0.28	0.880
	AH	3.83 ± 0.32^a^	4.73 ± 0.40a^b^	5.00 ± 0.29^b^	0.056
	*p*-value	0.129	0.587	0.098	
CK (U/L)	BH	98.00 ± 6.60	97.67 ± 5.02	97.33 ± 5.30	0.997
	AH	97.56 ± 6.62^a^	277.00 ± 24.15^b^	232.78 ± 28.52^b^	<0.001
	*p*-value	0.373	0.547	0.156	
LDH (U/L)	BH	495.33 ± 45.48	495.00 ± 33.08	496.78 ± 45.67	0.482
	AH	497.56 ± 43.18	538.56 ± 38.81	572.33 ± 47.17	0.999
	*p*-value	0.354	0.255	0.014	

Values are mean ± standard error, with different superscripts small letters, a, b, c--within a row differ significantly (*p* < 0.05), BH, value before-handling in lairage; AH, value after-handling at slaughter point; Control, goats moved from lairage to slaughter point gently; LI, goats moved from lairage to slaughter point by ear pulling; LP, goats moved from lairage to slaughter point by hindleg pulling; ALT, alanine aminotransferase; CK, creatine kinase; LDH, lactate dehydrogenase; level of significance- 5% (*p* < 0.05), *n* = 9.

The increase in blood glucose and lactate concentrations could be attributed to the higher increase in catecholamines and glucocorticoids under stress in goats. This resulted in increasing glucose production from glycogenolysis and gluconeogenesis required for preparing animals for the response to a stressor (12). Similar to the finding presented in this study, higher blood glucose levels were observed in Boer cross bucks by Kumar et al. (5), under psychological stress during the slaughtering of goats. Furthermore, an increase in blood glucose levels was observed to indicate stress in goats (35).

### 3.4 Blood enzymes (ALT, CK, and LDH)

The BH and AH plasma concentrations of ALT and CK of all three groups of goats did not change significantly upon preslaughter handling (Table 2). The LP goats had significantly (*p* = 0.014) higher AH value for LDH as compared to the BH value. For the BH groups, glucose, lactate, and CK values did not vary significantly. However, ALT and LDH values were recorded as comparable (*p* > 0.05) within the BH and AH groups. For the AH groups, glucose and lactate contents of treatment groups (EP and LP) were comparable and significantly higher than their respective control values. The AH LP and EP goats had significantly higher CK concentrations as compared to the AH control goats. The highest CK concentration was recorded for EP goats and the lowest for control groups, whereas LP exhibited intermediate values.

The muscle injury and inflammation due to preslaughter stress led to the increased synthesis of CK and LDH enzymes in the liver. Thus, CK and LDH prove good indicators of muscle injury and tissue damage caused under various preslaughter operations such as intense physical activity, rough handling, and trauma (36). The increased plasma concentration of CK and LDH was attributed to the increased cell permeability of cell membranes and increasing damage to the cells (36, 37). ALT is found in serum and organ tissue, especially in the liver. Its concentration is elevated in serum during myopathy, liver damage, congestive heart failure, and stress levels. In animals, ALT is also used as an indicator of adaptive capability and stress (38). Improper preslaughter handling of animals could lead to muscle injury and physical exertion in animals, consequently increasing the plasma concentration of LDH and CK (35, 39). The plasma ALT concentration recorded an increasing trend in goats under stressful conditions such as weaning stress (40), preslaughter transportation stress (41), and heat stress (42). Similar to the present study, increased plasma concentrations of CK in goats were observed (43) during preslaughter transportation stress and increased CK and LDH concentrations due to slaughter stress (44).

### 3.5 Hormonal responses

For LP, AH, β-endorphin values were significantly higher (*p* = 0.021) than BH (Table 3). The AH β-endorphin values in EP and control groups did not differ significantly from their respective BH values. The adrenaline concentration of LP goats AH was significantly (*p* = 0.045) higher than its BH value. There was no significant difference noted in this study in BH and AH adrenaline values among control groups and EP groups. Similarly, AH noradrenaline values for EP and LP goats were observed to be 2.12-fold and 1.94-fold higher than their respective BH values.

**Table 3:** Effects of pre-slaughter handling intensities on β- endorphin, adrenaline, and noradrenaline levels in goats.

Parameters	Sampling	Control	EP	LP	*p*-value
β- endorphin (pg/mL)	BH	71.26 ± 6.64	69.26 ± 6.69	68.11 ± 10.12	0.961
	AH	68.40 ± 4.28^a^	121.07 ± 32.96^a^	212.40 ± 32.47^b^	0.005
	*p*-value	0.276	0.688	0.021	
Adrenaline (pg/mL)	BH	68.60 ± 5.91	69.29 ± 5.60	66.23 ± 2.36	0.899
	AH	73.96 ± 5.40^a^	111.55 ± 12.56^b^	123.22 ± 11.28^b^	0.008
	*p*-value	0.554	0.614	0.045	
Noradrenaline (pg/mL)	BH	148.67 ± 16.11	152.95 ± 13.98	139.06 ± 10.89	0.771
	AH	168.59 ± 16.62	324.30 ± 67.83	271.05 ± 13.08	0.168
	*p*-value	0.247	0.033	0.870	

Values are mean ± standard error, with different superscripts small letters, a, b, c--within a row differ significantly (*p* < 0.05), BH, value before-handling in lairage; AH, value after-handling at slaughter point; Control, goats moved from lairage to slaughter point gently; EP, goats moved from lairage to slaughter point by ear pulling; LP, goats moved from lairage to slaughter point by hindleg pulling; level of significance- 5% (*p* < 0.05), *n* = 9.

Furthermore, noradrenaline concentration was comparable for the BH and AH in different groups (Table 3). BH, adrenaline and β-endorphin concentrations did not differ significantly between different groups. AH, β-endorphin concentration in LP goats was recorded as the highest and significantly higher than those of control and EP goats. Furthermore, after handling, the adrenaline concentration of EP and LP goats was recorded as comparable but significantly higher than that of control goats.

In the present study, the higher plasma catecholamines were recorded for EP and LP goats. This could be attributed to the high level of preslaughter stress in these goats. The preslaughter handling in the present study was for a short duration (5–7 min), thus activating the SPA system, consequently releasing catecholamines in the blood circulation for allostasis and catering to higher energy demands in stressful conditions (45). Similar to the present study, a higher catecholamine concentration was reported in goats under preslaughter transport stress (46), slaughter stress (44), and in lambs (35).

An increase in the plasma β-endorphin concentration was linked with stressful conditions rather than painful conditions in animals (9). Furthermore, the blood circulatory β-endorphin is not supposed to reach the central nervous system; thus, plasm β-endorphin is widely linked with stress rather than pain perception (47, 48).

## 4 Conclusion

The present study highlighted the importance of proper preslaughter handling in goats for mitigating stress, pain, and fear before slaughter and improving welfare compliance. The control goats handled gently with minimum human contact were observed to have lower behavioral, physiological, and hormonal responses, thereby indicating lower preslaughter stress and improved animal welfare status. To minimize detrimental effects on behavior, physiology, blood enzymes, and hormonal responses, it is advisable to adhere to animal welfare principles during preslaughter handling procedures. The goats should be handled gently and with minimum human contact to minimize stress and fear among goats during handling. The stockpersons should be properly trained in basic animal handling and behavioral principles to avoid improper handling of animals. Therefore, it is recommended to embrace animal welfare guidelines as a dependable strategy for achieving optimal preslaughter handling and avoid both ear and LP of goats.

## Data availability statement

The raw data supporting the conclusions of this article will be made available by the authors, without undue reservation.

## Ethics statement

The animal study was approved by Ethics Committee of Scientific Research, King Saud University (KSU), Saudi Arabia (Approval No: KSU-SE-22-112). The study was conducted in accordance with the local legislation and institutional requirements.

## Author contributions

AA-O: Data curation, Formal analysis, Funding acquisition, Methodology, Project administration, Resources, Writing – original draft, Writing – review & editing. GS: Conceptualization, Methodology, Project administration, Software, Supervision, Validation, Writing – original draft, Writing – review & editing. MA: Investigation, Software, Supervision, Writing – original draft, Writing – review & editing. AyS: Investigation, Methodology, Project administration, Supervision, Visualization, Writing – original draft, Writing – review & editing. MA-B: Data curation, Investigation, Software, Writing – original draft, Writing – review & editing. HB-A: Conceptualization, Investigation, Methodology, Writing – original draft, Writing – review & editing. AwS: Formal analysis, Supervision, Validation, Visualization, Writing – original draft, Writing – review & editing. PK: Investigation, Methodology, Writing – original draft, Writing – review & editing. UK: Data curation, Investigation, Writing – original draft, Writing – review & editing.
